# First Results From a Propensity Matching Trial of Mycophenolate Mofetil vs. Azathioprine in Treatment-Naive AIH Patients

**DOI:** 10.3389/fimmu.2021.798602

**Published:** 2022-01-11

**Authors:** George N. Dalekos, Pinelopi Arvaniti, Nikolaos K. Gatselis, Anna Samakidou, Stella Gabeta, Eirini Rigopoulou, George K. Koukoulis, Kalliopi Zachou

**Affiliations:** ^1^Department of Medicine and Research Laboratory of Internal Medicine, National Expertise Center of Greece in Autoimmune Liver Diseases, General University Hospital of Larissa, Larissa, Greece; ^2^Department of Pathology, Faculty of Medicine, School of Health Sciences, University of Thessaly, Larissa, Greece

**Keywords:** autoimmune hepatitis, autoimmune liver diseases, mycophenolate mofetil, azathioprine, immunosuppression, outcome

## Abstract

**Background/Aims:**

As previous real-world studies and meta-analyses have shown that mycophenolate mofetil (MMF) might have better efficacy than azathioprine (AZA) in autoimmune hepatitis (AIH), we conducted a propensity matching study to assess the efficacy and safety of MMF vs. AZA.

**Methods:**

All 126 consecutive treatment-naive adult AIH patients, diagnosed and followed in our department since 2016, were included. Patients received prednisolone 0.5–1 mg/kg/day plus either AZA 1–2 mg/kg/day or 1.5–2 g/day MMF. The tapering of prednisolone was identical between groups.

**Results:**

After propensity matching score and adjustment for known factors affecting response to treatment and outcome, 64 patients were included in the study (MMF = 32 and AZA = 32). Rates of non-response, complete biochemical response (CBR) at 6 and 12 months, and prednisolone withdrawal (6 months, 12 months, and end of follow-up) were identical between groups. However, MMF treatment was significantly associated with CBR at the end of follow-up [odds ratio (OR) 11.259; 95% CI: 1.3–97.4, p = 0.028]. AZA patients were more prone to stop treatment due to AZA intolerance/insufficient response (p = 0.0001). At the end of follow-up, the overall efficacy of each schedule was also significantly higher in the MMF group compared to the AZA group (p = 0.0001).

**Conclusion:**

We showed for the first time in a propensity matching study that MMF can be used as first-line therapy in AIH as attested by the significantly higher CBR at end of follow-up compared to AZA. Whether this better efficacy is also associated with higher histological remission rates and sustained CBR off immunosuppression needs further evaluation.

## Introduction

Autoimmune hepatitis (AIH) is a disease of unknown etiology characterized by distinct elevation of serum immunoglobulin G (IgG) levels even in patients without cirrhosis, female predominance, non-organ- and/or organ-specific autoantibodies, interface hepatitis on histology, and favorable response to immunosuppressive treatment (1–6). Administration of corticosteroids with or without azathioprine (AZA) is considered the standard of care as first-line therapy (2–6). The aims of such treatment are to achieve complete biochemical response (CBR; normal transaminases and IgG) and histological remission of the disease [modified hepatitis activity index (mHAI <4)] and, eventually, prevent fibrosis progression and development of end-stage liver disease at the minimal risk of side effects of therapy (2–6).

However, this strong recommendation is based on randomized trials mostly conducted in 1960s and 1970s, which however used diverse criteria of response, and when they were included in a systematic review, the response rate to standard treatment proved to be approximately 43% (7–15). Furthermore, in more recent studies and under real-world circumstances, it is now clear that approximately up to 40% of patients do not achieve CBR with standard treatment, and therefore, they are at risk of progressing to advanced disease (16–18). Furthermore, another study from UK with long-term follow-up showed that although these treatment modalities considerably reduced 5-year mortality rates, they failed to achieve histological remission and/or prevent fibrosis progression in many AIH patients (19). Supporting evidence for the problematic long-term efficacy of the standard conventional treatment come also from a large multicenter study from the Netherlands that showed that relapse of the disease is almost ubiquitous after AZA cessation, even though the patients were in long-term biochemical response (normal transaminases and IgG for more than 2 years before treatment discontinuation) (20).

AZA is metabolized to 6-mercaptopurine (6-MP) and subsequently in either 6-thioguanine (6-TGN) and 6-thiouric acid or 6-methyl mercaptopurine. It inhibits purine synthesis inducing non-selective immunosuppression, mainly through 6-TGN (21). However, thiopurine methyltransferase (TPMT; the enzyme that catalyzes the conversion of 6-MP to inactive metabolites) deficiency can skew 6-MP metabolism toward 6-TGN, leading to increased toxicity. In parallel, determination of TPMT activity or genotyping is not widely available in everyday clinical practice, while it seems that it cannot identify all patients who might develop AZA-related toxicity (22). Nevertheless, in a recent retrospective analysis, it has been shown that determination of AZA metabolite levels could improve biochemical response rates with fewer adverse events (17). Concisely, the standard treatment bares about 15%–25% rates of no response or intolerance to AZA, while patients who are not able to achieve predniso(lo)ne withdrawal and remained at doses higher than 10 mg/day for more than 18 months may suffer several corticosteroid-related complications (2–6). All together, these data could suggest that predniso(lo)ne with or without AZA is far from the ideal treatment option for AIH (15).

On the other hand, mycophenolate mofetil (MMF) is the prodrug of mycophenolic acid, which is activated after de-esterification by the liver. Mycophenolic acid is the first potent, selective, reversible, and non-competitive inhibitor of type-II isoform of inosine-5′-monophosphate dehydrogenase, leading to guanosine-triphosphate depletion specifically in activated B and T lymphocytes (23). This results in selective immunosuppression with few side effects, which is the requested aim in patients with autoimmune diseases or transplanted patients. In addition, we and others have shown in real-world prospective studies and in meta-analysis that MMF could be an alternative and safe first-line treatment option to induce and maintain response with a rapid steroid-sparing effect for patients with AIH (24–27). In addition, up to the present, these studies showed the highest rates of maintenance of remission after treatment withdrawal ever published for a median of 40 months off treatment in association with significant improvement of inflammatory activity and stable and/or improved fibrosis at second liver biopsy (24, 25). Based on these results, the Hellenic Association for the Study of the Liver (HASL) has included since early 2015 (https://www.eemh.gr/images/files/AIH_guidelines_06-04-2015.pdf), apart from AZA, MMF as an acceptable first-line treatment option for induction and maintenance of response in AIH patients (4). Accordingly, since 2016, we conducted a propensity matching trial of MMF vs. AZA administration in consecutive treatment-naive AIH patients in order to investigate if the previous results of the real-world uncontrolled studies on MMF efficacy are still of importance in the management of AIH patients after a face-to-face comparison.

## Patients and Methods

### Patients

Since March 2016, all 127 consecutive treatment-naive adult patients (≥16 years), who were diagnosed and followed in our department with well-established AIH according to the diagnostic criteria of the International AIH Group (IAIHG) (1) and were eligible for induction treatment according to the European Association for the Study of the Liver (EASL) (3) and HASL (4) clinical practice guidelines, were prospectively included in the study.

According to the 2015 HASL clinical practice guidelines, the patients decided to receive either combination therapy with prednisolone 0.5–1 mg/kg/day and MMF 1.5–2 g/day or the same prednisolone dose and AZA 1–2 mg/kg/day for at least 3 years (not more than 5 years). The exclusion criteria are shown in **Supplementary Table S1**. As it is shown in **Table 1**, the tapering schedule of prednisolone was identical between the two groups.

**Table 1:** The treatment protocol in the two groups of the study.

	Week	Prednisolone (0.5–1 mg/kg/day)	AZA group (n = 32) (1–2 mg/kg/day)	MMF group (n = 32, g/day)
Screening and consecutive propensity matching	0			
	1	Initial dose		1 g
	2	Initial dose		1 g
	3	Tapering 5 mg	50 mg	1.5 g
	4	Same dose as in week 3	50 mg	2 g
	5	Tapering 5 mg	75 mg	2 g
	6	Same dose as in week 5	75 mg	2 g
	7	Tapering 5 mg	100 mg	2 g
	8	Same dose as in week 7	100 mg	2 g
	9	Tapering 5 mg	150 mg	2 g
	10	Same dose as in week 9	150 mg	2 g
	11	Tapering 5 mg	150 mg	2 g
	12	Tapering 5 mg	150 mg	2 g
	13	Tapering 5 mg	150 mg	2 g
	14	Tapering 5 mg	150 mg	2 g
	15 and thereafter	Tapering 2.5 mg/week up to complete withdrawal	150 mg*	2 g**

*In sustained (>6 months) complete biochemical response off prednisolone, tapering of AZA (25 mg/6 months) up to the dose of 1 mg/kg/day.

**In sustained (>6 months) complete biochemical response off prednisolone, tapering of MMF (500 mg/year) up to the dose of 1 g/day.

AZA, azathioprine; MMF, mycophenolate mofetil.

In case of AZA intolerance and/or insufficient response during follow-up, treatment was switched from AZA to MMF. Intolerance was defined as any adverse event possibly related to treatment as assessed by treating physician leading to potential discontinuation of the drug (28). Insufficient response during follow-up was defined by sustained loss of CBR at any time during follow-up despite adherence to treatment and intensive immunosuppression.

In case of relapses during corticosteroid tapering or withdrawal, the patients received the same treatment schedule as at baseline (3, 4, 24, 25). Consideration for treatment withdrawal was done individually if the patient had received at least 3 consecutive years of immunosuppression being in CBR at least the last 2 years before discontinuation (3, 4, 25). A second liver biopsy before complete immunosuppression discontinuation was desirable but was not accepted by all patients. As we and others have described (29–31), in these cases, serial liver stiffness measurements (LSMs) using Fibroscan^®^ 502 (Echosens, Paris, France) equipped with the standard M probe performed to follow up changes of histological characteristics without liver biopsy.

The females of childbearing age were informed concerning the potential effect of teratogenicity, particularly in the MMF group, and were counseled for effective contraception during the whole study period and at least 6 months apart the potential drug withdrawal. All these patients should have at screening a negative pregnancy test, and they should be using or willing to apply birth control methods such as diaphragm, copper intrauterine device, condom by the partner, sponge, or spermicide and hormonal contraceptives.

All subjects provided written informed consent to participate in the study. The ethical committee of the General University Hospital of Larissa approved the study protocol that conforms to the ethical guidelines of the 1975 Declaration of Helsinki as revised in Brazil in 2013, as reflected in *a priori* approval by the institution’s human research committee (21-03-2016/2258).

### Autoantibodies

Antinuclear antibodies (ANA), smooth muscle antibodies (SMA), and liver kidney microsomal antibodies (anti-LKM) were initially detected by indirect immunofluorescence on 5-μm fresh frozen sections of in-house rodent multiorgan (kidney, liver, and stomach) substrates, with starting serum dilution at 1/40, according to the published guidelines (1, 4, 5) and our previous original reports (24, 25). Antibodies against soluble liver antigen/liver pancreas (anti-SLA/LP) and anti-LKM antibodies were also investigated by immunoblotting using rat cytosolic or liver microsomal extracts (24, 25, 32). Commercially available ELISA kits (INOVA, Diagnostics Inc., San Diego, CA, USA) using recombinant cytochrome P450 2D6 or SLA/LP/tRNP(Ser)Sec antigens were also used for anti-LKM and anti-SLA/LP detection, respectively, according to the manufacturer’s instructions.

### Primary and Secondary Treatment Endpoints

Primary endpoints were as follows:

- Non-response defined as <50% decrease of serum transaminases within 4 weeks after initiation of treatment (28)
- CBR (normal transaminases and IgG) at 6 and 12 months after treatment initiation and at the end of follow-up
- Prednisolone withdrawal rates
- Intolerance to treatment defined as any adverse event possibly related to treatment as assessed by the treating physician leading to potential discontinuation of the drug
- CBR after treatment changes
- CBR off treatment
- Histological remission of the disease defined as mHAI <4 at second liver biopsy

Secondary endpoints were as follows:

- The rapidity of achieving CBR at 6 months, 12 months, and at the end of follow-up
- The duration of CBR off prednisolone
- Relapses during tapering or withdrawal of corticosteroids
- Stable or improved liver disease at the histological level in second liver biopsy
- Changes after serial LSM determinations by transient elastography

### Safety Assessment

Safety was assessed by vital signs and physical examination in every visit along with follow-up investigation of the whole blood count and biochemical markers (**Supplementary Table S2**). All adverse events were encountered and characterized as serious or not and regimen-related or not.

### Statistical Analysis

Analysis was made using the SPSS 20 software. Results were expressed as median (range). In order to assess the response to treatment, we used the intention-to-treat (ITT) analysis. Accordingly, the missing data were dealt with using the last observation carried forward method through which the last available measurement for each subject at the time point prior to switch of treatment is retained in the analysis (33). We used the propensity score matching to compare patients between MMF and AZA groups. Propensity score was obtained by using logistic regression analysis including as covariates known factors that could affect the response to treatment and outcome (age, sex, the presence of cirrhosis at baseline, disease duration, seropositivity for anti-SLA/LP or anti-LKM, clinical severity of the disease, IgG levels, necroinflammatory activity, and fibrosis stage) (32, 34–36). Matching was performed 1:1 with the nearest-neighbor method within caliper bounds of ±0.2 (37, 38). To address potential confounding bias, we investigated the effect of the type of treatment on response to treatment using the binary logistic regression analysis. Data were compared using Mann–Whitney U-test for the detection of differences between independent samples and Wilcoxon test for paired samples, while chi-square and Fisher’s exact tests were applied for detecting differences between categorical variables. Two-sided p*-*values <0.05 were considered as statistically significant in 95% confidence interval.

## Results

### Propensity Matching and Baseline Characteristics of Autoimmune Hepatitis Patients

According to the exclusion criteria (**Supplementary Table S1**), 11 patients were excluded because they were receiving only prednisolone and another 2 because they did not receive any treatment due to burned-out cirrhosis. In addition, 4 patients (1 under MMF and 3 under AZA treatment) were excluded because of the presence of AIH/primary biliary cholangitis variant and 6 patients (4 under MMF and 2 under AZA treatment) because of AIH/primary sclerosing cholangitis variant. After the implementation of propensity score 1:1 matching in the remaining 104 patients, 64 patients were included in the study (32 patients on MMF and 32 patients on AZA). The characteristics of the patients who could not be properly matched with each other are shown in **Supplementary Table S3**.

As shown in **Table 2**, the baseline demographics, clinical, laboratory, serological, and histological characteristics of the patients did not significantly differ between the two groups. At the time of this analysis, the total follow-up of the patients was 39 (7–70) months and the total duration of treatment up to the last follow-up was 36.5 (6–67) months. In addition, the median starting dose of prednisolone was 60 (20–125) mg/day in the MMF group and 50 (25–75) mg/day in the AZA group (p = 0.112); the median prednisolone dose at the time of this analysis was 0 (0–35) mg/day and 0 (0–20) mg/day, respectively.

**Table 2:** Baseline demographics, clinical, laboratory, serological, and histological characteristics in the two groups of the study.

Characteristics	MMF (n = 32)	AZA (n = 32)	p
Sex (female/male)	23/9	23/9	NS
Age at diagnosis (years)	54 (15–80)	55 (15–84)	NS
Time to diagnosis (months)	2.5 (1–402)	3 (1–142)	NS
Acute presentation	16	16	NS
Insidious/asymptomatic presentation	16	16	NS
Follow-up (months)	45 (12–63)	38 (7–70)	NS
Total treatment duration (months)*	39 (11–63)	34 (6–67)	NS
Concurrent autoimmune diseases	18/32	11/32	NS
AIH revised diagnostic score	18 (11–24)	17 (11–22)	NS
AIH simplified diagnostic score	7 (6–8)	7 (6–9)	NS
ALT (IU/ml, ULN: 40)	412 (43–3,716)	425 (8–1,843)	NS
Bilirubin (mg/dl, ULN:1.1)	2 (0.5–13.4)	1.4 (0.3–14.3)	NS
γ-GT (IU/L, ULN: 40)	101 (16–1,136)	159 (14–902)	NS
IgG (mg/dl, ULN: 1,500)	1,844 (782–3,240)	1,760 (870–3,740)	NS
Anti-SLA/LP**	5/32	5/32	NS
Anti-LKM	0/32	0/32	NS
ANA**	17/32	19/32	NS
SMA**	31/32	29/32	NS
Cirrhosis at diagnosis (yes/no)	6/26	6/26	NS
mHAI activity score	8 (3–16)	7 (3–14)	NS
mHAI fibrosis score	2 (2–6)	1 (1–5)	NS

Data are expressed as median (range).

Abbreviations are the same as in the text.

*Denotes the duration of treatment from initiation until the last follow-up.

NS, not statistically significant; γ-GT, gamma-glutamyl transpeptidase; ULN, upper limit of normal.

**All patients had at least one autoantibody positive.

### Response to Treatment and Treatment Changes During Follow-Up Because of Insufficient Response and/or Intolerance to Initial Treatment

During follow-up, patients receiving standard treatment were more prone to stop treatment because of insufficient response [3/32 (9%)] and/or intolerance [9/32 (28.1%)] compared to MMF-treated patients (12/32 vs. 0/32, respectively; p = 0.0001). Patients with intolerance to AZA were treated with MMF according to guidelines (3, 4), while patients with insufficient response were also switched to MMF as second-line treatment.

**Figure 1:**
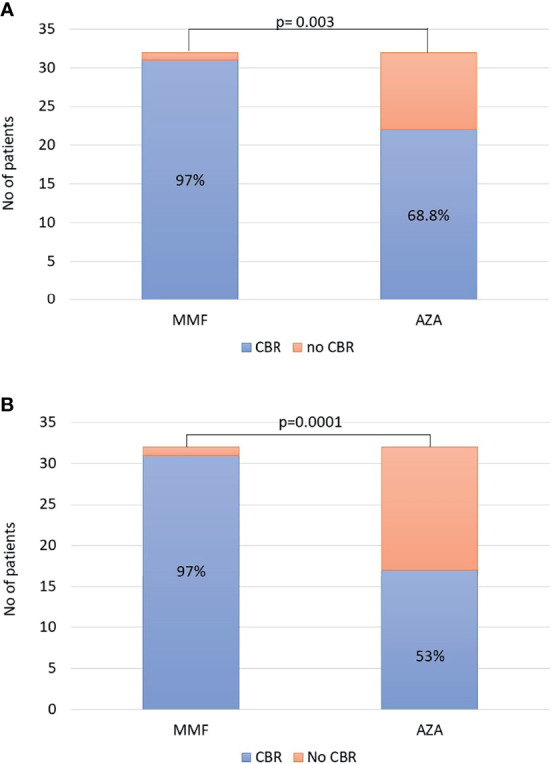
**(A)** Intention-to-treat analysis: complete biochemical response (CBR) at last follow-up was significantly higher in the mycophenolate mofetil (MMF) group [31/32 (97%)] compared to that in the azathioprine (AZA) group [22/32 (68.8%); p = 0.003]. **(B)** At the end of follow-up, the overall efficacy of each schedule was significantly higher in the MMF group [31/32 (97%)] compared to that in the AZA group [17/32 (53%); p = 0.0001].

The non-response rates (week 4 of treatment) did not significantly differ between the two groups [2/32 (6.2%) in the MMF group vs. 4/32 (12.5%) in the AZA group]. Since patients treated with AZA started it after being 14 days on prednisolone monotherapy, we also compared non-response rate on week 4 of treatment for the MMF group and on week 6 of treatment for the AZA group. However, there was also no significant difference between the groups [2/32 (6.2%) in the MMF group vs. 2/32 (6.2%) in the AZA group]. In ITT analysis, the CBR rates in patients on MMF and AZA at 6 and 12 months did not differ [6 months: 30/32 (93.8%) vs. 26/32 (81%) and 12 months: 30/32 (93.8%) vs. 25/32 (78%), respectively]. However, CBR at last follow-up was significantly higher in the MMF group (31/32; 97%) compared to the AZA group (22/32; 68.8%; p = 0.003; **Figure 1A**).

Moreover, after using the binary logistic regression model considering the type of treatment (MMF vs. AZA) and the propensity score, treatment with MMF proved to be significantly associated with CBR at the end of follow-up compared to standard treatment [odds ratio (OR) 11.259; 95% CI: 1.3–97.4, p = 0.028] (**Table 3**). On the contrary, binary logistic regression showed that the type of treatment did not affect response to treatment at months 6 and 12 (p > 0.05 for both; data not shown).

**Table 3:** Binary logistic regression for estimating the effect of type of treatment on response to treatment.

Variables in the Equation									
		B	SE	Wald	df	Sig.	Exp(B)	95% CI for EXP(B)	
								Lower	Upper
Step 1^a^	MMF_vs_AZA (1)	2,421	1,101	4,835	1	0,028	11,259	1,301	97,433
	Propensity_score	-2,581	2,605	0,981	1	0,322	0,076	0,000	12,494
	Constant	2,251	1,547	2,119	1	0,146	9,500		

^a^Variable(s) entered on step 1: MMF_vs_AZA, Propensity_score.

The rates of corticosteroid withdrawal between the MMF and AZA groups were not different at 6 months [5/32 (15.6%) vs. 3/32 (9.3%), respectively, p = 0.7], 12 months of treatment [14/32 (43.8%) vs. 12/32 (37.5%), respectively, p = 0.6], and at the end of follow-up [26/32 (81%) vs. 19/32 (59.4%), respectively; p = 0.10]. Logistic regression considering the propensity score showed similar results for 6 and 12 months and end of follow-up (p > 0.05 for all). The rapidity of corticosteroid cessation was also identical [11 (4–55) vs. 9 (3–26) months, respectively; p = 0.319]. Furthermore, the cumulative prednisolone dose did not differ between the two groups (341.3 ± 279.6 vs. 341.6 ± 286.2, respectively; p > 0.05). In addition, the duration of CBR off corticosteroids was not different between the two groups [24.5 (1–58) months; n = 26 vs. 19 (1–52) months; n = 19, respectively].

### Analysis of Patients on Azathioprine Who Switched to Mycophenolate Mofetil and Overall Analysis

All 12 patients who switched from AZA to MMF achieved CBR after switching at the end of follow-up. The duration of treatment with MMF in this group (n = 12) was 19 (5–47) months.

Apart from the ITT analysis, at the end of follow-up, the overall efficacy of each schedule was also significantly higher in the MMF group (31/32, 97%) compared to that in the AZA group (17/32, 53%; p = 0.0001; **Figure 1B**) . The duration of this CBR was 39 (11–63) months in the MMF group (n = 31) and 38 (6–67) months in the AZA group (n = 17) (p = 0.76).

Relapses during tapering or withdrawal of corticosteroids were observed in 8/32 (25%) in the MMF group and 11/32 (34%) in the AZA group (p > 0.05).

### Histological Remission at Second Liver Biopsy and Complete Biochemical Response Off Treatment

At the time of this writing, 5 patients who were eligible for stopping treatment according to the EASL and HASL guidelines withdrew treatment. Four of them underwent a second liver biopsy and stopped treatment (2 in the MMF and 2 in the AZA group). The fifth patient (AZA group) did not consent to a second liver biopsy, but his LSM had improved (from 17.3 to 5.7 kPa; **Table 4**), and therefore, he also stopped treatment. The time of second liver biopsy is shown in **Table 4**. Inflammation improved in all, while fibrosis also improved or remained stable in all (**Table 4**). The CBR off treatment for each patient is also shown in **Table 4**. In addition, to date, all 5 patients maintain remission for 6 (6–9) months.

**Table 4:** Liver histology data in 5 autoimmune hepatitis patients who stopped treatment.

Patient No.	Treatment	First biopsy (baseline)	Second biopsy (months)	Second biopsy	Prednisolone (second biopsy)	ALT (IU/L, ULN: 40)	IgG (mg/dl, ULN: 1,500)	Fibroscan (first biopsy)	Fibroscan (second biopsy)
1	MMF	mHAI: 9	55	mHAI: 3	No	15	810	7.4 kPa	4.8 kPa
		mHAI stage: 2		mHAI stage: 2					
2	MMF	mHAI: 7	36	mHAI: 2	No	17	1,109	12 kPa	5.7 kPa
		mHAI stage: 1		mHAI stage: 1					
3	AZA	mHAI:10	59	mHAI: 3	No	32	1,190	4.5 kPa	3.5 kPa
		mHAI stage:1		mHAI stage: 2					
4	AZA	mHAI:4	62	mHAI: 2	No	19	1,040	3.4 kPa	4.4 kPa
		mHAI stage:3		mHAI stage: 3					
5	AZA	mHAI: 9	60	na	No	14	1,210	17.3 kPa	5.7 kPa
		mHAI stage: 2		na					

MMF, mycophenolate mofetil; AZA, azathioprine; mHAI, modified Hepatitis Activity Index; na, not applicable; ALT, alanine aminotransferase; IgG, immunoglobulin G; ULN, upper limit of normal.

### Safety Issues

MMF was well tolerated (up to the present, none of the MMF-treated patients discontinued treatment), while switching to MMF was observed in 9/32 (28.1%) of AZA-treated patients because of intolerance (p = 0.0006). Mild gastrointestinal symptoms were reported in 2 patients from the MMF and 3 from the AZA group. These side effects were temporary and did not need dose reduction or hospitalization in both arms. During follow-up, 2 patients under MMF treatment developed lower respiratory tract infection, 1 had varicella zoster virus reactivation, and 1 presented cellulitis. No patient needed hospitalization. However, in all of these four patients, MMF was temporarily discontinued for 1–2 weeks and then treatment was readministered progressively until maximum dose (2 g/day) with no further complications. In the AZA group, 1 patient developed a severe lower respiratory tract infection and another 1 suffered from an episode of severe herpes simplex virus stomatitis. Admission to the hospital was needed in both patients, and therefore, treatment was subsequently switched to MMF after complete recovery. No patient in the MMF group developed myelotoxicity. In contrast, 2 patients from the AZA group developed leukopenia and 1 thrombocytopenia, and therefore, treatment was changed to MMF. Moreover, 4 patients in the AZA group presented moderate to severe increases of transaminases with no simultaneous elevation of IgG levels, which was attributed to AZA hepatotoxicity, and treatment was switched to MMF. Up to the present, none of the females of childbearing age became pregnant in both arms.

## Discussion

Herein we present the first results from an open-label prospective propensity matching study that investigated the safety and efficacy of MMF vs. AZA in treatment-naive patients with AIH who received induction and maintenance therapy for 3–5 years. To the best of our knowledge, this is the first study that assessed face-to-face MMF vs. AZA as first-line treatment option in AIH patients. The median follow-up of the patients was more than 3 years, so some safe conclusions can be drawn. In addition, in this study, the new definitions of response criteria and endpoints proposed by the IAIHG were evaluated (28). Up to the present, the following three major points arise from the present investigation: 1) patients receiving AZA were significantly more prone to stop treatment because of insufficient response and/or intolerance; 2) at the end of follow-up, the ITT analysis showed that the CBR rate was significantly higher in the MMF-treated patients compared to that in the AZA group; and 3) apart from the ITT analysis, at the end of follow-up, the overall efficacy of each schedule was also significantly higher in the MMF group compared to that in the AZA group, even though the rates of corticosteroid withdrawal and the rapidity of this cessation were identical between the two groups.

This study confirmed the results of our two previous prospective real-world observational studies (24, 25) published 10 and 5 years ago, as well as the study by Hlivko et al. (26) who reported 84% response rate in 29 AIH patients treated with MMF including 17 treatment-naive patients and a recent meta-analysis where the combination of prednisone with MMF as first-line treatment proved to enable AIH patients to obtain significantly higher CBR rates and a lower non-response rate compared to standard treatment (27). So far, MMF has been evaluated in retrospective studies but in more instances only as second-line rescue therapy in patients with AIH who were either unresponsive or intolerant to AZA. Indeed, a recent study from the Australian Liver Association Clinical Research Network confirmed that MMF is an excellent treatment option for AIH patients either intolerant or refractory to standard treatment with those most likely to respond being older at MMF initiation or with lower international normalized ratio or IgG levels at baseline (39), although current recommendations suggest that patients intolerant to AZA have more benefit compared to those with insufficient response (3, 4). In another recent systematic review with meta-analysis (40), an overall high efficacy of MMF as second-line treatment in AIH was reported with low discontinuation rates, which is in accordance with the results of our previous (24, 25) and the present study.

Considering tolerability, similar to our previous studies (24, 25), the present propensity matching trial showed a safe profile of MMF, as it has also been reported in patients with systemic lupus erythematosus or other autoimmune diseases as well as in transplant recipients (41, 42). Up to the present, none of the patients in the MMF group suffered serious side effects, and therefore, none of them discontinued or switched therapy to AZA. This is in accordance with our previous studies (24, 25) and with most of the previous studies that used MMF as salvage treatment for AIH (39, 40). In contrast, 28% of patients in the AZA group suffered from intolerance to the drug, which is in accordance with the published rates of AZA intolerance in the literature. Of interest, all 12 patients who shifted to MMF achieved CBR. However, it has to be underlined that MMF should be given under strict contraceptive measures in females of reproductive age, as, in contrast to AZA, it is absolutely contraindicated in pregnancy (43). Another potential drawback regarding MMF use is the issue of cost. Of course, MMF is more expensive than AZA; however, the use of MMF generics as in our case minimizes this disadvantage while not only the direct cost should be considered. For instance, according to the guidelines, the routine laboratory tests are performed more often in the AZA-treated patients compared to MMF, while there were no admissions or day offs due to side effects in the MMF group.

Unfortunately, up to the present, because of the type of this study, we are not able to see if this better efficacy of MMF is also associated with higher rates of histological remission and sustained CBR off treatment. Indeed, so far, 7.8% of the total number of patients were eligible to stop treatment according to the EASL and HASL guidelines (6.3% in MMF-treated patients and 9.4% in the AZA group, p > 0.05). This important question will be addressed soon after the long-term results of the study.

Our study has some limitations, as this is not a randomized trial that in turn may raise concerns for potential bias. Indeed, such bias cannot be completely excluded. However, the patients received prospectively MMF or AZA in a consecutive manner, and then for the analysis, we used the strict propensity matching score, taking into consideration not only the age and sex but also several other known factors that could affect response to treatment. As a result, we believe that our propensity matching study minimizes the potential bias and increases the reliability of our findings.

In conclusion, we showed for the first time in a face-to-face comparison that MMF-treated patients with AIH achieved significantly higher rates of CBR compared to that of standard treatment. Whether these first results are also associated with higher rates of remission at the histological level and sustained CBR after immunosuppression cessation needs to be addressed in the forthcoming years after the long-term results of the study. In addition, after these first positive results from the propensity matching trial, a randomized trial under the auspices of HASL comparing again MMF vs. AZA in AIH patients is scheduled to start in the country during the first half of next year.

## Data Availability Statement

The original contributions presented in the study are included in the article/**Supplementary Material**. Further inquiries can be directed to the corresponding author.

## Ethics Statement

The studies involving human participants were reviewed and approved by ethical committee of the General University Hospital of Larissa. The patients/participants provided their written informed consent to participate in this study.

## Author Contributions

Study concept and design: GD and KZ. Acquisition of research data: PA, NG, AS, SG, and ER. Analysis and interpretation of data: PA, AS, KZ, NG, GK, and GD. Drafting of the article: GD, PA, KZ, and NG. Critical revision and editing of the article: GD, KZ, NG, ER, and GK. All authors contributed to the article and approved the submitted version.

## Conflict of Interest

The authors declare that the research was conducted in the absence of any commercial or financial relationships that could be construed as a potential conflict of interest.

## Publisher’s Note

All claims expressed in this article are solely those of the authors and do not necessarily represent those of their affiliated organizations, or those of the publisher, the editors and the reviewers. Any product that may be evaluated in this article, or claim that may be made by its manufacturer, is not guaranteed or endorsed by the publisher.
