# Supplementary material for: First Results From a Propensity Matching Trial of Mycophenolate Mofetil vs. Azathioprine in Treatment-Naive AIH Patients
Source: Front Immunol. 2022 Jan 11;12:798602. doi: 10.3389/fimmu.2021.798602 (PMC8787111; doi:10.3389/fimmu.2021.798602)
Supplement: Supplementary file 1 [file Table_1.docx]

**Supplementary Table 1.** Exclusion criteria of the study.

| Diagnosis of AIH/primary biliary cholangitis or AIH/primary sclerosing cholangitis variant |
| --- |
| Baseline neutrophils less than 1500/μL |
| Compensated or decompensated burn-out cirrhosis |
| Pregnancy or unwillingness to take effective contraception measures |
| Hepatocellular cancer, spontaneous bacterial peritonitis, hepato-renal syndrome, hepatic encephalopathy, or refractory ascites |
| Active extra-hepatic malignancy or active alcoholism |
| Chronic renal failure under hemodialysis or with estimated glomerular filtration rate <30 ml/min |
| Active infection, sepsis or current gastrointestinal hemorrhage |
| Recent (<30 days) major surgery e.g. for cancer, coronary artery disease, valve replacement or total hip replacement |
| Current treatment with prednisolone or other immunomodulatory drugs |
| Patients having treated with any biological therapy the last 6 months |
| Known hypersensitivity to the drugs used in the protocol |
| Primary immunodeficiency disorders |
| Human immunodeficiency virus infection |
| Unwillingness to consent or active psychosis |
