# Supplementary material for: First Results From a Propensity Matching Trial of Mycophenolate Mofetil vs. Azathioprine in Treatment-Naive AIH Patients
Source: Front Immunol. 2022 Jan 11;12:798602. doi: 10.3389/fimmu.2021.798602 (PMC8787111; doi:10.3389/fimmu.2021.798602)
Supplement: Supplementary file 2 [file Table_2.docx]

**Supplementary Table 2.** Schedule of visits and blood testing.

| **Visits** | **Screening** | **Baseline** | **W3** | **W6** | **W12** | **W18** | **W24** | **W36** | **W48** | **W64** | **W80** | **W96** | **W120** | **W144** | **W168** | **W192** | **W216** | **W240** | **W260** |
| --- | --- | --- | --- | --- | --- | --- | --- | --- | --- | --- | --- | --- | --- | --- | --- | --- | --- | --- | --- |
| Consent | **Χ** |  |  |  |  |  |  |  |  |  |  |  |  |  |  |  |  |  |  |
| Strict and detailed propensity matching | **Χ** |  |  |  |  |  |  |  |  |  |  |  |  |  |  |  |  |  |  |
| Prednisolone initiation |  | **X** |  |  |  |  |  |  |  |  |  |  |  |  |  |  |  |  |  |
| ΑΖΑ initiation |  |  | **X** |  |  |  |  |  |  |  |  |  |  |  |  |  |  |  |  |
| MMF initiation |  | **X** |  |  |  |  |  |  |  |  |  |  |  |  |  |  |  |  |  |
| Detailed history | **Χ** |  |  |  |  |  | **X** |  | **X** |  |  | **X** |  | **X** |  | **X** |  |  | **X** |
| Physical examination | **Χ** |  | **X** | **X** | **X** | **X** | **X** | **X** | **X** | **X** | **X** | **X** | **X** | **X** | **X** | **X** | **X** | **X** | **X** |
| Lab testing | **Χ** | **Χ** | **Χ** | **X** | **X** | **X** | **X** | **X** | **X** | **X** | **X** | **X** | **X** | **X** | **X** | **X** | **X** | **X** | **X** |
| Pregnancy test | **Χ** |  |  |  |  |  | **X** |  | **X** |  |  | **X** |  | **X** |  | **X** |  |  | **X** |
| Questionary | **Χ** |  |  |  |  |  | **X** |  | **X** |  |  | **X** |  | **X** |  | **X** |  |  | **X** |
| Safety | **X** | **Χ** | **Χ** | **X** | **X** | **X** | **X** | **X** | **X** | **X** | **X** | **X** | **X** | **X** | **X** | **X** | **X** | **X** | **X** |
