# Supplementary material for: First Results From a Propensity Matching Trial of Mycophenolate Mofetil vs. Azathioprine in Treatment-Naive AIH Patients
Source: Front Immunol. 2022 Jan 11;12:798602. doi: 10.3389/fimmu.2021.798602 (PMC8787111; doi:10.3389/fimmu.2021.798602)
Supplement: Supplementary file 3 [file Table_3.docx]

Supplementary Table 3. Baseline demographics, clinical, laboratory, serological and histological characteristics of the 40 patients who could not be matched according to their propensity score (calliper 0.2).

| **Characteristics** | **MMF (n=29)** | **AZA (n=11)** | **P** |
| --- | --- | --- | --- |
| Sex (female/male) | 19/10 | 7/4 | NS |
| Age at diagnosis (years) | 54 (33-80) | 46 (20-72) | 0.025 |
| Time to diagnosis (months) | 27 (0-360) | 5 (1-50) | NS |
| Acute presentation | 18/29 | 2/11 | 0.034 |
| Follow-up (months) | 28 (6-68) | 26 (8-48) | NS |
| Total treatment duration (months)* | 25 (5-82) | 19 (7-103) | NS |
| Disease duration (months) | 57 (16-375) | 21 (14-128) | 0.045 |
| Concurrent autoimmune diseases | 17/29 | 2/11 | 0.053 |
| AIH revised diagnostic score | 18 (10-22) | 17 (13-22) | NS |
| AIH simplified diagnostic score | 6 (6-9) | 6 (6-8) | NS |
| ALT (IU/mL, ULN: 40) | 460 (44-4925) | 190 (43-1108) | NS |
| Billirubin (mg/dl, ULN:1.1) | 0.95 (0.27-27.9) | 1 (0.3-13.1) | NS |
| γ-GT (IU/L, ULN: 40) | 64 (14-541) | 92 (10-1327) | NS |
| IgG (mg/dL, ULN: 1500) | 1870 (1090-2710) | 2229 (1153-3990) | 0.048 |
| Anti-SLA/LP** | 9/29 | 0/11 | 0.043 |
| Anti-LKM** | 3/29 | 0/11 | NS |
| ANA** | 10/29 | 6/11 | NS |
| SMA** | 25/29 | 10/11 | NS |
| Cirrhosis at diagnosis (yes/no) | 9/29 | 0/11 | 0.043 |
| mHAI activity score | 8 (3-15) | 5 (3-10) | 0.098 |
| mHAI fibrosis score | 3 (1-6) | 1 (1-5) | 0.037 |

Data is expressed as median(range). Abbreviations are same as in the text. *Denotes the duration of treatment from initiation till the last follow-up. NS, not statistically significant; γ-GT, gamma-glutamyl transpeptidase; ULN, upper limit of normal. **All patients had at least one autoantibody positive
